# Two-layer accumulated quantized compression for communication-efficient federated learning: TLAQC

**DOI:** 10.1038/s41598-023-38916-x

**Published:** 2023-07-19

**Authors:** Yaoyao Ren, Yu Cao, Chengyin Ye, Xu Cheng

**Affiliations:** 1 School of Information and Control Engineering, Liaoning Petrochemical University, Fushun, Liaoning, People’s Republic of China; 2 School of Economics and Management, Shenyang Agricultural University, Shengyang, Liaoning, People’s Republic of China

**Keywords:** Computational science, Computer science

## Abstract

Federated learning enables multiple nodes to perform local computations and collaborate to complete machine learning tasks without centralizing private data of nodes. However, the frequent model gradients upload/download operations required by the framework result in high communication costs, which have become the main bottleneck for federated learning as deep models scale up, hindering its performance. In this paper, we propose a two-layer accumulated quantized compression algorithm (TLAQC) that effectively reduces the communication cost of federated learning. TLAQC achieves this by reducing both the cost of individual communication and the number of global communication rounds. TLAQC introduces a revised quantization method called RQSGD, which employs zero-value correction to mitigate ineffective quantization phenomena and minimize average quantization errors. Additionally, TLAQC reduces the frequency of gradient information uploads through an adaptive threshold and parameter self-inspection mechanism, further reducing communication costs. It also accumulates quantization errors and retained weight deltas to compensate for gradient knowledge loss. Through quantization correction and two-layer accumulation, TLAQC significantly reduces precision loss caused by communication compression. Experimental results demonstrate that RQSGD achieves an incidence of ineffective quantization as low as 0.003% and reduces the average quantization error to 1.6 × ${10}^{-5}$. Compared to full-precision FedAVG, TLAQC compresses uploaded traffic to only 6.73% while increasing accuracy by 1.25%.

## Introduction

With the rapid advancements in emerging technologies like the Internet of Things (IoT) and edge computing, the volume of data generated at the network's edge has been growing exponentially. A significant amount of valuable data is distributed across different terminal devices. Traditional deep learning methods typically require central storage of training data, which poses challenges in achieving centralized integration of data in natural environments. This situation leads to the formation of "data islands" and creates barriers between data sources. In 2016, Google introduced the concept of federated learning specifically designed for mobile devices. Federated learning^1,2^ emerged as a solution to address the problem of data islands to some extent. McMahan et al.^3^ described the federated learning framework for deep learning tasks and proposed the well-known FedAVG algorithm (Federated Averaging Algorithm). The key aspect of federated learning is that it eliminates the need to share private data among nodes, granting nodes complete control over their locally stored data. In a typical worker-server architecture^4–8^, worker nodes upload their local model training information (such as gradients or parameter updates) to a central server. The server utilizes the uploaded information from worker nodes to update the global model using an aggregation algorithm.

However, on the one hand, federated learning requires a large number of communications between nodes to achieve good model accuracy; On the other hand, with the continuous increase in the scale of deep learning, the number of model parameters has exploded, which sharply increases the cost per communication of federated learning. Limited by network conditions and bandwidth, the limitation of communication cost prevents many edge nodes from participating in federated learning. High communication cost has become the main bottleneck of federated learning. To address the challenge of high communication costs in federated learning, researchers have proposed various communication compression methods aimed at reducing communication overhead in both federated learning and distributed machine learning. These methods aim to alleviate the burden of communication while maintaining or improving the overall performance of the federated learning process.

Neural network pruning has been an earlier method proposed for model compression and acceleration. Srinivas et al.^9^ used pruning methods to remove redundant neurons, and Han et al.^10,11^ reduced the total number of parameters and operations for the entire network, removed redundant connections, and quantified parameters. Vanhoucke et al.^12^ showed that 8-bit quantization of parameters could significantly speed up models with minimal loss of accuracy. The primary objective of model compression is to alleviate storage and computational burdens on nodes, but it also indirectly reduces communication costs by reducing network complexity and parameter size. Later, quantization methods were directly applied to study communication-efficient distributed machine learning. In contrast to model compression, communication compression focuses solely on improving communication efficiency. Predecessors have proposed various gradient quantization methods^13–19^ to accelerate data-parallel distributed learning by quantifying the communication content. Inspired by the concept of model delay update^21,22^, Storm et al.^23^ introduced gradient sparsification method by only sending gradients that exceed a certain threshold. Subsequently, Aji, Yin, et al.^24,28^ proposed different strategies for gradient selection. In contrast to the aforementioned gradient sparsification approach, Chen et al.^27^ treat the gradients of the entire node as the minimum unit for gradient selection, meaning that a node either sends all subgradients or does not send any at all. This sparse method reduces the number of communication rounds and is referred to as the communication sparsification method. Both quantization and sparsification schemes significantly reduce the communication cost of distributed machine learning. In the context of communication-efficient federated learning, the research focus lies in reducing the impact of gradient loss on model convergence under high compression. The goal is to achieve effective compression while maintaining satisfactory model convergence during the federated learning process.

In our research, we observed that the QSGD^14^ (Quantized Stochastic Gradient Descent) algorithm^14^, when applied to deep learning, exhibits an ineffective quantization phenomenon, leading to inefficient communication. To address this issue, we propose the TLAQC (Two-Layer Accumulated Quantized Compression) algorithm, which improves upon the predecessor's quantization algorithm. Additionally, we incorporate the communication sparsification method to further reduce communication costs. Moreover, we introduce a two-layer accumulation approach to compensate for the loss incurred by communication compression. TLAQC demonstrates high practicality and can be flexibly applied to distributed machine learning and federated learning scenarios. The main contributions of this paper are summarized as follows:

(1)when the quantization algorithm QSGD^14^ is applied to deep learning, a large number of values are quantized to 0.In this paper, we refer to the phenomenon of zeroing out non-zero gradient values during deep model training as ineffective quantization. Severe ineffective quantization issues result in unnecessary communication waste, and zero gradient values do not contribute to model training. In this regard, this paper introduces the RQSGD (Revised QSGD) algorithm, which builds upon QSGD and incorporates zero-value correction into the quantization process. This additional cost introduces only a minimum factor of full precision.Simulation experiments demonstrate that RQSGD significantly reduces the ratio of quantization error and ineffective quantization compared to QSGD, thereby improving quantization efficiency.

(2)To further reduce the communication cost, TLAQC combines sparsification method with quantization. We deduce the global adaptive threshold and model parameter self-inspection formula. Worker nodes utilize the adaptive threshold and self-inspection formula to perform parameter self-inspection on the quantized model parameters. Nodes that do not meet the threshold criteria skip the current communication round, thereby achieving the goal of sparse communication.

(3)For worker nodes that successfully pass the threshold check, the quantization error of the current round is locally recorded. On the other hand, for nodes that fail the check, the weight deltas of the corresponding training are stored locally. In the subsequent training round, the quantization error and retained model weight deltas are accumulated to minimize the loss caused by communication compression.

## Ralated works

### Federated learning

**Figure 1:**
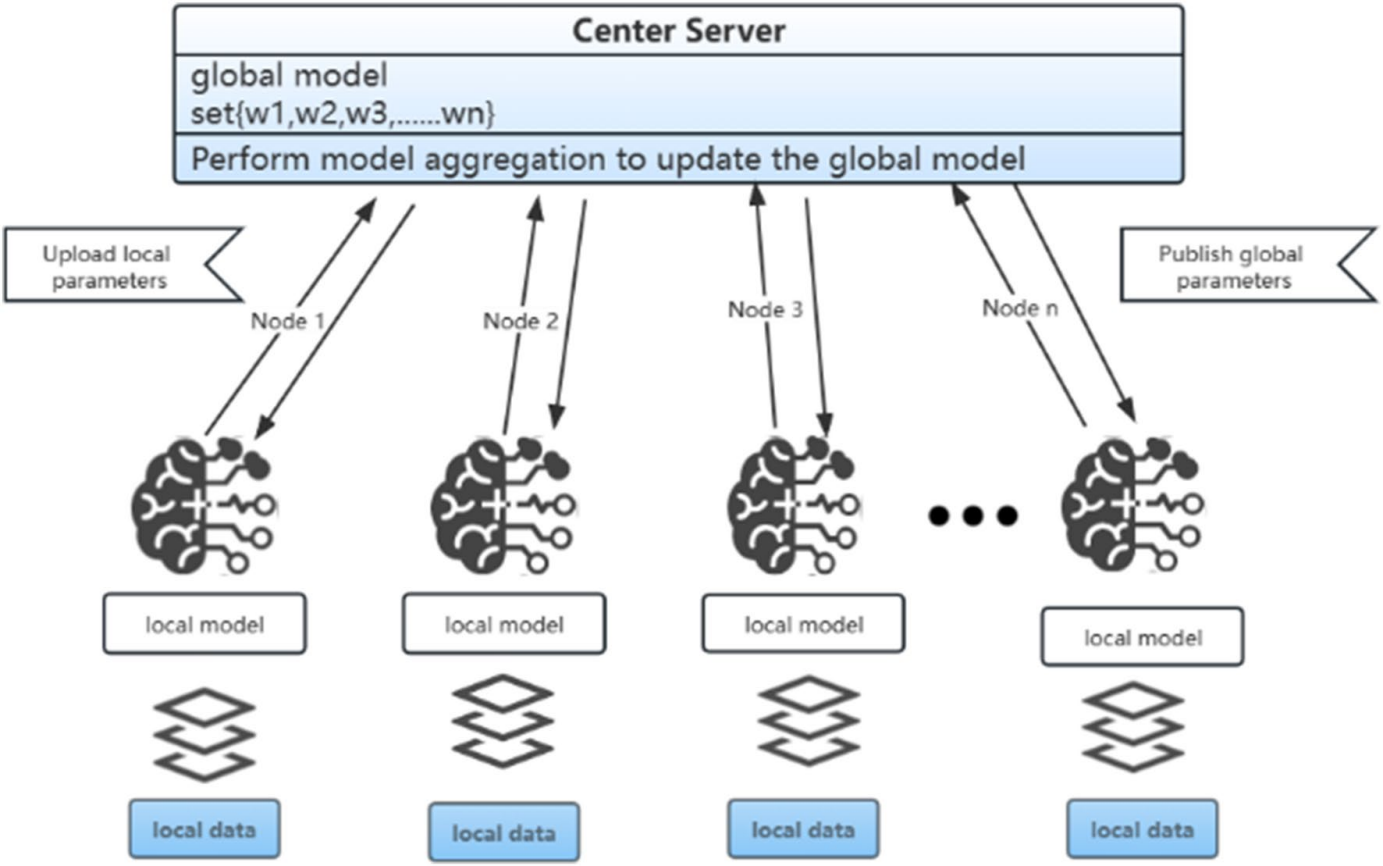
Federated learning under server-worker architecture.

Federated learning is a specific type of distributed machine learning, where the training data or models are divided among multiple training nodes to form a computational cluster. This approach utilizes multiple computing devices to collaboratively perform machine learning tasks. The parameter server architecture^4–8^ is a widely adopted architecture in distributed machine learning. It consists of worker nodes and central parameter servers, where worker nodes perform parallel computations on different data to compute weight deltas, and the server updates global parameters based on the uploaded information from the workers. This architecture follows a typical master–slave configuration, as illustrated in Fig. 1. The FedSGD algorithm is designed based on the parameter server architecture, where all worker nodes need to upload and download parameters with the central parameter server in each training round. To enhance the efficiency of federated learning, FedAVG, an extension of FedSGD, proposes performing multiple local iterations of SGD on the nodes before uploading the local computation results to the central node. Consequently, FedAvg is regarded as a communication-efficient federated learning algorithm.

Assuming that the set of all worker nodes participating in the training task is M, the goal of the optimization problem is:

1 $$\min_{w \in W} \mathop \sum \limits_{p \in M} \mathop \sum \limits_{{x_{i} \in D_{p} }} f\left( {w;\;x_{i} } \right)$$

where, $W$ is the parameter space, $f$ is the objective function. ${D}_{p}$ is the local dataset of worker p, and the server updates the global model according to:

2 $$\Theta_{global}^{k} = \mathop \sum \limits_{p \in M} \omega_{p} w_{p}^{k - 1}$$

${w}_{p}^{k-1}$ is the parameters updated locally by the worker *p* based on the global model parameters ${\theta }_{global}^{k-1}$ of the k − 1 round, and ${\omega }_{p}$ is the parameter weight value of the worker p (the dataset size of the worker p accounts for the total dataset size). When N workers have the same data size, and the information uploaded by the worker nodes is the weight deltas ∆w, the function mentioned above can be transformed into the following function:

3 $$\Theta_{global}^{k} = \Theta^{k - 1} + \frac{1}{\left| M \right|}\mathop \sum \limits_{p \in M} \Delta w_{p}^{k - 1}$$

### Quantization

Gradient quantization refers to reducing the communication traffic by reducing the accuracy of the gradient. Most modern computers use 32 or 64 bits to represent a floating-point number, and quantization reduces the number of digits represented by each value, thereby compressing traffic several times, which is extremely obvious in Deep Learning. Representing the original value with a low number of bits will inevitably lead to quantization errors, and the focus of quantization methods is on how to reduce quantization errors and the damage of quantization errors to model convergence. Seide et al.^13^ proposed 1-bit quantization, which only retains the sign of the gradient, and reduces the impact on the convergence speed by adding quantization error back to the residential gradient. Alistarh et al.^14^ proposed the QSGD algorithm and proved the convergence of the algorithm. It trades between convergence and quantization levels and reduces the communication cost by adjusting the number of bits sent. Wen et al.^15^ quantized the floating-point number to {− 1, 0, + 1}, proposed the TernGrad algorithm, and gave proof of convergence under the assumption that the gradient is bounded. He et al.^20^ believed that the gradients with larger magnitudes are more critical, and proposed a nonlinear quantization algorithm CosSGD based on the cosine function so that the value with more significant gradients has a finer quantization space.

### Sparsification

Gradient sparsification refers to selectively sending partial gradients and reducing communication costs by discarding some gradients with small contributions. The study found that the gradients calculated by nodes in distributed SGD are often sparse, and most gradient values are close to 0. Such gradients exchange are not only redundant but also increase the communication cost. The redundancy of gradients exchange provides theoretical feasibility for sparsifying gradients, and how to select gradients is the focus of the sparsification schemes. Storm et al.^23^ proposed a method to select gradients according to a pre-set threshold, and the workers send the gradients to the central parameter server only when the gradients are greater than the specified threshold. However, Machine Learning and Deep Learning models are rich and diverse, and the datasets are also very different, so it is difficult to fix an appropriate threshold in advance. In this regard, Aji et al.^24^ proposed to use a fixed compression rate (compressed size/pre-compressed size) to select the sent gradients; Dryden et al.^25^ propose sparsifying gradients using fixed proportions of positive and negative gradients; Hardy et al.^26^proposed an adaptive compression algorithm (AdaComp), which sorts the gradient values, selects the largest k items for transmission, and considers the influence of the decay effect of the gradient on model training. Chen^27^ et al. proposed a sparse communication algorithm LAG, which adaptively calculates a threshold in each round of training, aiming to skip part of the communication that transmits gradients. Unlike the methods above, the LAG algorithm reduces the communication cost in the federated learning mechanism by reducing the communication frequency between the worker nodes and the central parameter server.

## Two-layer accumulated quantized compression

This section provides a detailed explanation of the TLAQC (Two-Layer Accumulated Quantized Compression) algorithm proposed in this paper. TLAQC algorithm primarily consists of quantization and communication sparsification methods. In Section "RQSGD", we introduce a revised quantization method called RQSGD (Revised QSGD) to address the issue of ineffective quantization. Building upon RQSGD compression, Section "Commuinication Sparsification" reduces the frequency of workers uploading local information through communication sparsification, thereby further enhancing the level of communication compression. The two-layer accumulation refers to the accumulation of quantization errors and skipped weight deltas at the workers to compensate for the accuracy loss caused by communication compression. It is worth noting that in the context of this paper, if M represents a set, |M| denotes the cardinality of the set M. Similarly, if x represents a value, |x| represents the absolute value of x, and sgn(x) represents the sign of x. Furthermore, if v represents a vector, $\Vert v\Vert$ represents the ${l}_{2}$ or ${l}_{\infty }$ norm of the vector v.

### RQSGD

**Figure 2:**
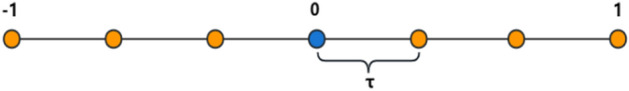
Quantization space (b = 3).

By quantizing full-precision floating-point numbers, the communication cost of distributed and federated learning can be significantly reduced. The quantization process of RQSGD consists of two steps. In the first step, for a given vector $v\in {R}^{n}$, performing b-bit quantization on the $i$-th entry of v, the quantization operation ${\widetilde{Q}}_{b}\left({v}_{i}\right)$ in RQSGD is similar to QSGD^14^ and is defined as follows (Fig. 2):

4 $$\tilde{Q}_{b} \left( {v_{i} } \right) = \left\| v \right\|_{\infty } \cdot sgn\left( {v_{i} } \right)\cdot\xi _{b} \left( {\left\| v \right\|_{\infty } ;\left| {v_{i} } \right|} \right)$$

where ${\Vert v\Vert }_{\infty }$ represents the scaling factor. We define $\tau :=1/({2}^{b-1}-1)$, and ${\xi }_{b}({\Vert v\Vert }_{\infty };\left|{v}_{i}\right|)$ maps $\left|{v}_{i}\right|$ to the quantized space { 0,τ,2τ,⋯,1}:

5 $$\xi _{b} \left( {\left\| v \right\|_{\infty } ;\left| {v_{i} } \right|} \right) = \left\{ {\begin{array}{*{20}c} {l\tau ,\,\,\,~with~probability~1 + l - \frac{{\left| {v_{i} } \right|}}{{\tau \left\| v \right\|_{\infty } }}~~} \\ {\left( {l + 1} \right)\tau ,~~~otherwise~~~~~~~~~~~~~~~~~~~~~~~~~~~~~~~~~~~~~} \\ \end{array} } \right.$$

$\left|{v}_{i}\right|/{\Vert v\Vert }_{\infty }$ falls in the interval $\left[l\tau ,\left(l+1\right)\tau \right]$, $l$ is an integer between $\left[0,\right.\left.{2}^{b-1}-1\right)$. Denote the quantization error of ${v}_{i}$ by ${\varepsilon }_{i}$, $\left|{\varepsilon }_{i}\right|\le \Vert v\Vert \cdot \tau /2$. Unlike QSGD, the scaling factor of ${\widetilde{Q}}_{b}\left({v}_{i}\right)$ uses ${l}_{\infty }-norm$ instead of ${l}_{2}-norm$. $\left|{v}_{i}\right|/{\Vert v\Vert }_{\infty }$ can more evenly distribute the values in the $\left[0,\right.\left.1\right]$ interval, that is, more evenly distributed in the quantization space. Equation (4) is easy to quantize the smaller values in the vector to 0, while the vast majority of model gradients or weight deltas in Deep Learning are close to 0, which makes the right side of the quantization space underutilized. Suppose δ represents a certain weight deltas vector, $\delta \in {R}^{n}$, if b = 2, scaling factor ‖δ‖ = x, and 99% of the value ${\delta }_{i}$ in δ satisfies $\left|{\delta }_{i}\right|/x<1/2$, then δ 99% of the values in will be quantized to 0. In deep learning, zero-valued gradients are not beneficial for model training. To address the phenomenon of quantizing non-zero gradient values to zero in deep learning, we propose the concept of ineffective quantization.The transmission of a large number of zero values not only hampers the convergence speed of the model but also leads to significant communication waste.In this context, RQSGD addresses this issue by correcting the quantized zero values. Formula (6) demonstrates this correction process, which involves using a full-precision value to record the minimum absolute value in the current quantization vector.

6 $$Q_{b} \left( {v_{i} } \right) = \left\{ {\begin{array}{*{20}l} {{\text{sgn}} \left( {v_{i} } \right)\cdot\min \left( {abs\left( v \right)} \right),} \hfill & {if \tilde{Q}_{b} \left( {v_{i} } \right) = 0} \hfill \\ {\tilde{Q}_{b} \left( {v_{i} } \right),} \hfill & {else} \hfill \\ \end{array} } \right.$$

**Figure 3:**
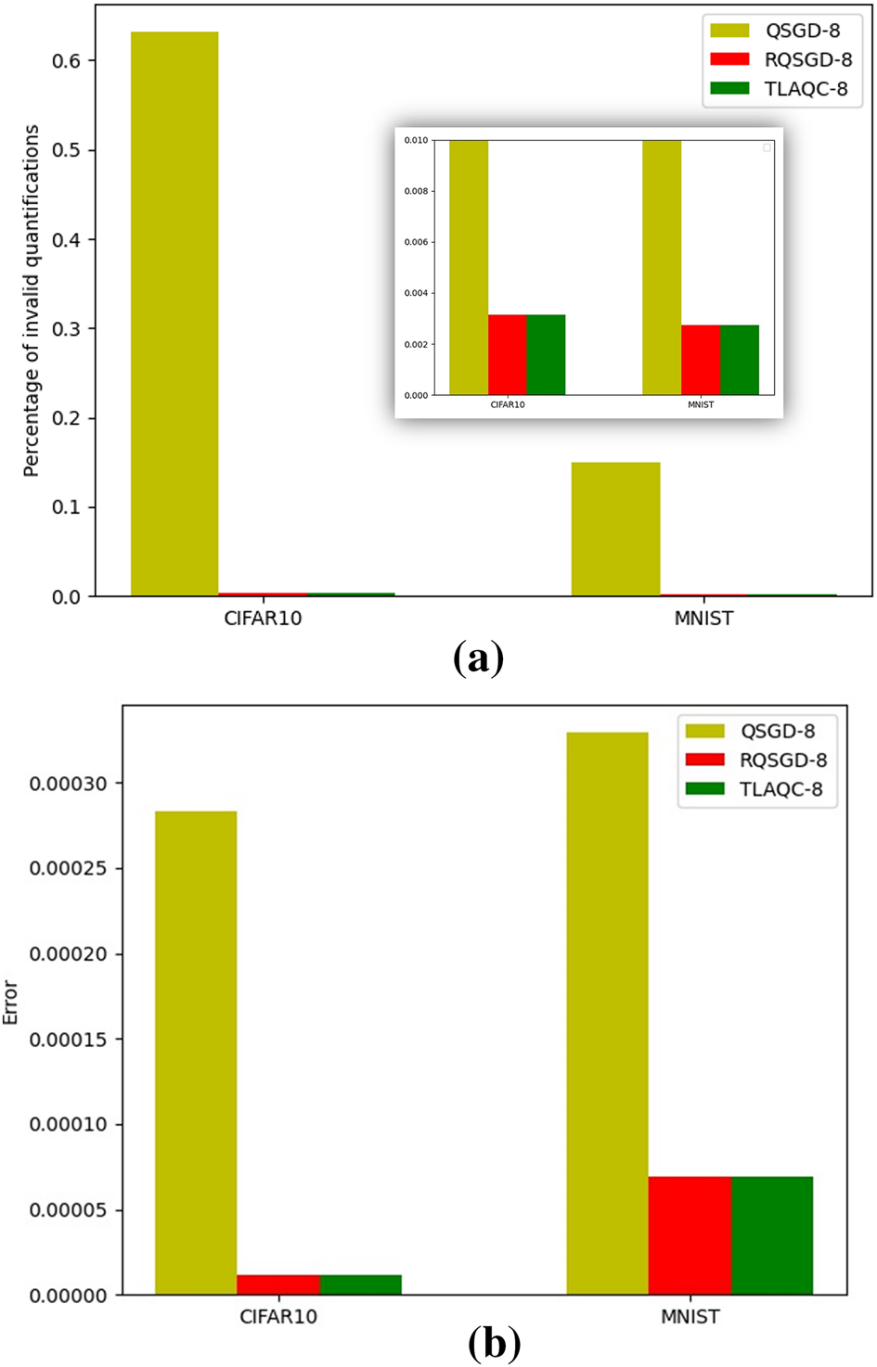
(**a**): The proportion of ineffective quantification; (**b**): Average quantization error for weight deltas.

We refer to $\mathrm{min}\left(abs(v)\right)$ as the minimum factor. Formula (6) utilizes $\mathrm{min}\left(abs(v)\right)$ to transform the quantized zero-valued gradient values in the vector, thereby mitigating the phenomenon of ineffective quantization. Ineffective quantization results in a large number of values being quantized to zero in the vector. By applying the minimum factor transformation to the quantized zero values, it helps reduce the quantization error of model parameters and the number of gradient values quantized to zero (Fig. 3).

For an n-dim vector v, if the number of full-precision bits is 32 bits, RQSGD quantizes the size of 32n bits to 64 + nb bits, which includes a full-precision scaling factor and minimum factor, b < 32. Figure 2 illustrates an implementation of RQSGD using a quantization level of b = 3.

The QSGD algorithm quantizes all model parameters by dividing them into vectors of the same dimension. We also adopted this approach in some of our experiments. However, when dealing with larger deep learning models, this approach introduces excessive computational overhead and communication costs. To address the quantization of deep learning models, we have observed that the model parameters within the same layer tend to have a more similar distribution. Therefore, for training larger models, we have opted for layer-wise quantization as it is more suitable.we uses ∆w to represent the weight deltas and defines $\Delta {w}_{p}^{k}:={w}_{p}^{k}-{\theta }^{k}$, ${\theta }^{k}$ represents the global model parameters of the k-th round, ${w}_{p}^{k}$ represents the model parameters of the worker p after the k-th round of training. For the quantization error generated by RQSGD, it is accumulated locally at the node:

7 $$e_{p}^{k} = \Delta w_{p}^{k} + \alpha e_{p}^{k - 1} - Q_{b} \left( {\Delta w_{p}^{k} + \alpha e_{p}^{k - 1} } \right)$$

M represents the set of workers, ${e}_{p}^{0}$=0, k ≥ 1, then the iteration of the global model is as follows:

8 $$\theta^{k + 1} = \theta^{k} + \sum\nolimits_{p = 1}^{\left| M \right|} {\frac{{\left| {D_{p} } \right|}}{\left| D \right|}Q_{b} \left( {\Delta w_{p}^{k} + \alpha e_{p}^{k - 1} } \right)}$$

where $\left|{D}_{p}\right|/\left|D\right|$ represents the model aggregation weight of worker p, $\left|{D}_{p}\right|$ represents the amount of data contributed by worker p, |D| represents the sum of the amount of data contributed by all workers, and α is the time decay weight of quantization error (0 ≤ α ≤ 1).

### Commuinication Sparsification

In the context of the high communication cost in federated learning, various gradient sparsification methods have been proposed. Previous approaches used fixed thresholds^28^ or fixed ratios^24^ as communication criteria to reduce the communication traffic between workers and the central server. However, these fixed conditions have limited scalability and struggle to adapt to changes in gradient values during training. To address this issue, this paper introduces an adaptive threshold parameter self-inspection module that extends gradient sparsification to communication sparsification through worker parameter self-inspection. Gradient sparsification means that all nodes upload a portion of their local gradient information, while communication sparsification selects certain nodes to upload all their gradient information. In TLAQC, the adaptive threshold dynamically adjusts with each training round, automatically adapting to the changing gradients and demonstrating excellent scalability. The parameter self-inspection formula is described below.

Assuming that the set of all workers participating in the training task is M, the set of workers sending parameters in the k-th round is ${M}_{s}^{k}$, and the set of workers not sending parameters is ${M}_{r}^{k}$, then the following formula is satisfied:

9 $$\frac{{\mathop \sum \nolimits_{{p \in M_{r}^{k} }} \left\| {Q_{b} \left( {\Delta w_{p}^{k} + \alpha e_{p}^{{k - 1}} } \right)} \right\|_{2}^{2} }}{{\left| {M_{r}^{k} } \right|}} \le \frac{{\mathop \sum \nolimits_{{p \in M}} \left\| {Q_{b} \left( {\Delta w_{p}^{k} + \alpha e_{p}^{{k - 1}} } \right)} \right\|_{2}^{2} }}{{\left| M \right|}}$$

If each node $i$ in the set ${M}_{r}^{k}$ satisfies the formula (10), then the above formula (9) must be satisfied:

10 $$\left\| {Q_{b} \left( {\Delta w_{i}^{k} + \alpha e_{i}^{{k - 1}} } \right)} \right\|_{2}^{2} \le \frac{{\mathop \sum \nolimits_{{p \in M}} \left\| {Q_{b} \left( {\Delta w_{p}^{k} + \alpha e_{p}^{{k - 1}} } \right)} \right\|_{2}^{2} }}{{\left| M \right|}}$$

In order to measure the contribution made by node $p$ in the k-th round of training, the following definition is given:

11 $$norm_{p}^{k} { \sim } = \left\| {Q_{b} \left( {\Delta w_{p}^{k} + \alpha e_{p}^{{k - 1}} } \right)} \right\|_{2}^{2}$$

Then the average contribution made by the k-th round of workers is:

12 $$norm\_avg^{k} { \sim } = \frac{{\mathop \sum \nolimits_{{p \in M_{s}^{k} }} norm_{p}^{k} }}{{\left| {M_{s}^{k} } \right|}}$$

In the k-th round of training, it is difficult for the worker to know the ${norm}_{p }^{k}$ value of other n-1 workers, so it is impossible to get the value of ${norm\_avg}^{k}$, so the norm_avg of the previous d rounds is used to approximate it instead, then the global adaptive threshold of the k-th round of training is:

13 $$threshold^{k} { \sim } = \frac{1}{d}\mathop \sum \limits_{j = 1}^{d} norm\_avg^{k - j}$$

The parameter self-inspection expression is: ${norm}_{i}^{k}\le {threshold}^{k}$, namely:

14 $$\left\| {Q_{b} \left( {\Delta w_{i}^{k} + \alpha e_{i}^{{k - 1}} } \right)} \right\|_{2}^{2} \le \frac{1}{{d\left| {M_{s}^{{k - j}} } \right|}}\mathop \sum \limits_{{j = 1}}^{d} \mathop \sum \limits_{{p \in M_{s}^{{k - j}} }} \left\| {Q_{b} \left( {\Delta w_{p}^{{k - j}} + \alpha e_{p}^{{k - j - 1}} } \right)} \right\|_{2}^{2}$$

where d is a hyperparameter that determines the number of previous rounds used to approximate the current round threshold. The right side of Eq. (14) represents the expression for the adaptive threshold. When the value of ${norm}_{i}^{k}$ calculated by worker i exceeds the threshold, which is computed and sent to the worker nodes by the central server, worker i initiates communication with the central server. On the other hand, if the ${norm}_{i}^{k}$ value is below the threshold, communication is reserved and no transmission occurs.

### TLAQC

By integrating RQSGD and the communication sparsification method, TLAQC achieves substantial improvements in the communication efficiency of federated learning. To address the accuracy loss resulting from communication compression, TLAQC incorporates a two-layer accumulation approach, where quantization errors and sparsified communication information are accumulated locally on the worker nodes. Section "RQSGD" has provided an initial explanation of the quantization error, and the subsequent section will delve deeper into the two-layer accumulation mechanism of TLAQC.

After the worker node p updates the model using its local dataset, it checks the recorded value of the quantization error ${e}_{p}^{k}$. If the quantization error is non-zero, the weighted quantization error is accumulated into the updated model parameters. Similarly, the worker checks the recorded value of the accumulated weight deltas ${h}_{p}^{k}$. If the worker did not communicate with the central server in the previous round, the weighted cumulative weight deltas are accumulated into the updated model.Once the two-layer accumulation is completed, the worker utilizes the accumulated model parameters to perform quantization calculations. The quantized parameters are then used to calculate the ${norm}_{p}^{k}$ value using the following calculation method:

15 $$\Delta w_{p}^{k} = w_{p}^{k} - \Theta_{global}^{k}$$

If worker p ∈ $${M}_{s}^{k}$$,

16 $$e_{p}^{k} = \Delta w_{p}^{k} + \alpha e_{p}^{k - 1} + \beta h_{p}^{k - 1} - Q_{b} \left( {\Delta w_{p}^{k} + \alpha e_{p}^{k - 1} + \beta h_{p}^{k - 1} } \right)$$

17 $$h_{p}^{k} = 0$$

If worker p ∈ $${M}_{r}^{k}$$,

18 $$e_{p}^{k} = 0$$

19 $$h_{p}^{k} = \Delta w_{p}^{k} + \alpha e_{p}^{k - 1} + \beta h_{p}^{k - 1}$$

where t represents the number of rounds since worker p last communicated with the central server. The global model update method in TLAQC is as follows:

20 $$\begin{aligned} \theta^{k + 1} = & \theta^{k} + \mathop \sum \limits_{{p \in M_{s}^{k - 1} \cap M_{s}^{k} }} \frac{{\left| {D_{p} } \right|}}{\left| D \right|}Q_{b} \left( {\Delta w_{m}^{k} + \alpha e_{p}^{k - 1} } \right) \\ & + \mathop \sum \limits_{{p \in M_{r}^{k - 1} \cap M_{s}^{k} }} \frac{{\left| {D_{p} } \right|}}{\left| D \right|}Q_{b} \left( {\Delta w_{m}^{k} + \beta h_{p}^{k - 1} } \right) \\ \end{aligned}$$

After incorporating two-layer accumulation , formulas (11) and (14) were rewritten as follows:

21 $$norm_{p}^{k} { \sim } = Q_{b} \left( {\Delta w_{p}^{k} + \alpha e_{p}^{k - 1} + \beta h_{p}^{k - 1} } \right)_{2}^{2}$$

22 $$\left\| {Q_{b} \left( {\Delta w_{i}^{k} + \alpha e_{i}^{{k - 1}} + \beta h_{p}^{{k - 1}} } \right)} \right\|_{2}^{2} \le \frac{1}{{d\left| {M_{s}^{{k - j}} } \right|}}\mathop \sum \limits_{{j = 1}}^{d} \mathop \sum \limits_{{p \in M_{s}^{{k - j}} }} \left\| {Q_{b} \left( {\Delta w_{p}^{{k - j}} + \alpha e_{p}^{{k - j - 1}} + \beta h_{p}^{{k - 1}} } \right) - } \right\|_{2}^{2}$$

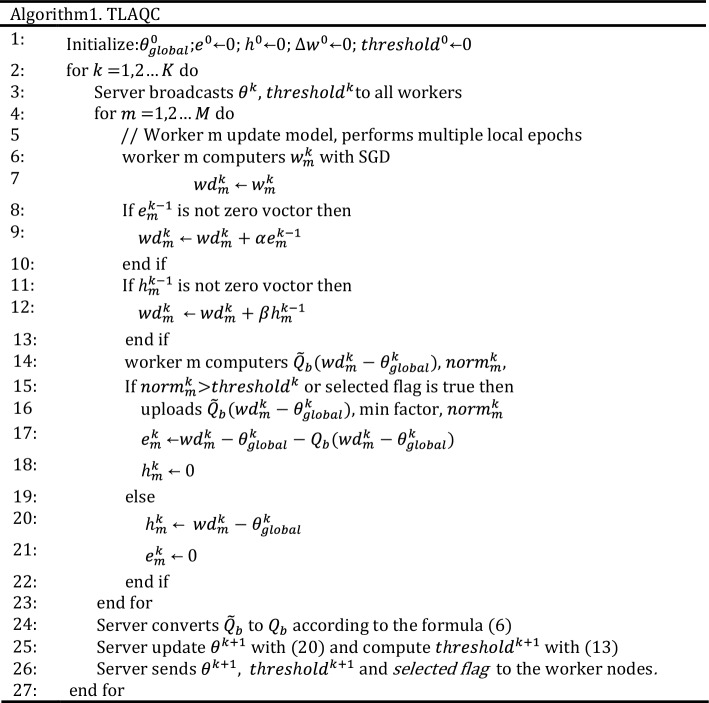

## Experiment results

### Settings

Based on the aforementioned description of the TLAQC algorithm, we will evaluate its performance using a convolutional neural network model in deep learning and two public datasets. In distributed communication compression algorithms, the degree of communication compression is a critical metric for measuring performance. Therefore, we employ the communication compression rate (cr) as a measure of the communication compression performance:

$$cr = \frac{{{\text{Number~}}\;{\text{of}}\;{\text{~bits~}}\;{\text{uploaded~}}\;{\text{after}}\;{\text{~communication~}}\;{\text{compression}}}}{{{\text{Number~}}\;{\text{of~}}\;{\text{bits}}\;{\text{~uploaded~}}\;{\text{before~}}\;{\text{communication~}}\;{\text{compression}}}} \times 100\%$$

In the experiments evaluating the aforementioned algorithms, two datasets were used: MNIST for handwritten digit recognition and CIFAR10 for object recognition. MNIST consists of ten categories ranging from 0 to 9, with a training set of 60,000 grayscale images of size 28 × 28 and a testing set of 10,000 images. For this experiment, the training set was randomly divided into 100 parts, each containing 600 images, with 10 parts assigned as the local dataset for 10 workers. Upon receiving the global parameters distributed by the central server, each worker utilizes stochastic gradient descent to update the global model. The local model configurations are as follows: local epochs = 5, batchsize = 64, lr = 0.01. The local training model employs a convolutional neural network architecture with three convolutional layers and two fully connected layers. The global training consists of 100 epochs, with a time decay weight α = 0.8, β = 0.8, and d = 1. The model parameters are divided into vectors of the same dimension for quantization, with a length of 512.

The CIFAR10 dataset consists of 32 × 32 colored images, including 50,000 training and 10,000 testing image samples, with a total of 10 categories. For this experiment, the training set is randomly divided into 10 parts, and each data set has 5000 images as the local training data of ten workers. The configuration of the local model is as follows: local epochs = 5, batchsize = 64, lr = 0.0005, weight_decay = 0.005. The local training model consists of 3 VGG-type blocks and two fully connected layers. Each block consists of two 3 × 3 convolutional layers, followed by a max pooling layer, a regularization layer, and a dropout layer. The global training consists of 150 epochs, with a time decay weight α = 0.8, β = 0.8, and d = 10. This part adopts a layer-wise quantization approach.

All experiments in this paper employ the SGD optimizer with a momentum of 0.9 and utilize the cross-entropy loss function. For the purpose of fair comparison, in the final round of global training, all members' contributions are aggregated. This means that in the last training round, each worker does not perform parameter self-inspection. Additionally, to update the global model parameters in each round of training, one worker is randomly selected for direct aggregation without parameter self-inspection.

### Parameter Study

For the hyperparameters α and β in RQSGD and TLAQC, we conducted simulation experiments on the MNIST dataset to obtain relatively optimal parameter settings. Table 1 presents the training results of RQSGD with an error compensation mechanism under different values of α.

**Table 1:** Comparision of RQSGD on MNIST with different $\boldsymbol{\alpha }$.

∝	Accuracy
1	98.32
0.9	98.35
**0.8**	**98.36**
0.7	98.29
0.6	98.28
0.5	93.31
0.4	98.30
0.3	98.30
0.2	98.28
0.1	98.27

Significant are in value 0.8.

Based on the results in Table 1, we set α = 0.8 for TLAQC. Table 2 presents the results of parameter tuning for β.

**Table 2:** Comparision of TLAQC on MNIST with different β.

∝	Accuracy
1	98.29
0.9	98.36
**0.8**	**98.39**
0.7	98.27
0.6	98.29
0.5	98.28
0.4	98.25
0.3	98.23
0.2	98.25
0.1	98.22

Significant are in value 0.8.

According to the simulation results, it was found that when α = 0.8 and β = 0.8, the model achieved relatively higher accuracy. When α is set too small, the effect of error feedback is not significant, and when β is set too small, the information loss caused by communication sparsity noticeably affects the algorithm's accuracy. On the other hand, if α and β are set too large, the outdated gradient effect can impact the convergence of the algorithm.

### Results

In this section, we assess the performance of our proposed algorithm compared to several prominent algorithms on the MNIST and CIFAR10 datasets. We consider full-precision FedAVG as the baseline algorithm and compare our algorithm to QSGD^14^ and CosSGD^20^, both utilizing 8-bit quantization. Additionally, we evaluate our algorithm against the communication sparsification algorithm LAG.

The Fig. 3 illustrates a comparison between the revised quantization algorithm RQSGD and QSGD during the deep learning training process, aiming to mitigate the issue of ineffective quantization and reduce quantization errors. The "proportion of ineffective quantification" refers to the ratio of the number of gradients quantized to zero throughout the entire model training process. The "proportion of ineffective quantification" represents the average quantization error of each parameter throughout the entire model training process. The calculation method for the "proportion of ineffective quantification" is as follows: the sum of the number of ineffective quantization generated by all workers/(number of model parameters × ${\sum }_{k=1}^{global epoch}\left|{M}_{s}^{k}\right|$). The calculation method for the "proportion of ineffective quantification" is as follows: the sum of quantization errors generated by all workers/(number of model parameters × ${\sum }_{k=1}^{global epoch}\left|{M}_{s}^{k}\right|$). The results show that RQSGD exhibits a significant improvement over QSGD in addressing ineffective quantization, resulting in reduced quantization errors during the training process.

**Figure 4:**
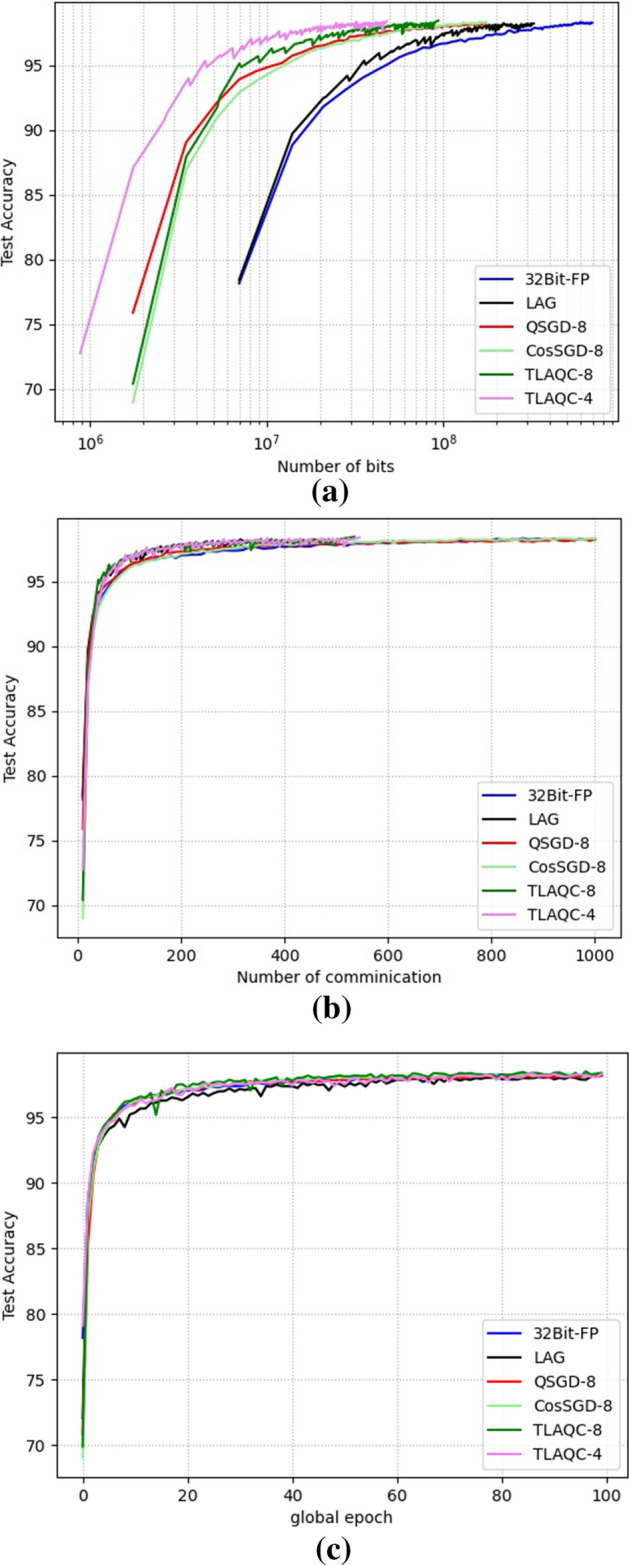
Comparision on MNIST ((**a**) accuracy versus bits; (**b**) accuracy versus communication; (**c**) accuracy versus global epoch).

The Fig. 4 presents the convergence behavior of TLAQC. When considering the same bit transmission, TLAQC demonstrates superior convergence performance, with TLAQC-4 exhibiting the fastest convergence speed. Under the same number of communication rounds, communication sparsification algorithms TLAQC and LAG converge more rapidly, with TLAQC outperforming LAG. In terms of the same number of training rounds, TLAQC-8 demonstrates better convergence performance, while TLAQC-4 closely follows TLAQC-8 in the training process.

It can be observed that due to the varying number of workers aggregated in each round, the convergence curve of communication sparsification algorithms exhibits noticeable oscillations before convergence. For instance, in the k-th round of global training, if there are too few aggregation nodes, the accuracy for that round may significantly drop. In the k + 1 round, the nodes that did not participate in the previous round's aggregation tend to generate a higher ${norm}^{k+1}$ value, increasing the probability of passing the parameter self-inspection and subsequently participating in the global aggregation for that round. This leads to a substantial improvement in accuracy.

Table 3 presents the algorithm performance of TLAQC and various comparison algorithms with the same number of global training rounds. TLAQC achieves significantly higher accuracy compared to full-precision FedAVG. Among the TLAQC variants, TLAQC-8 exhibits the best convergence accuracy, while TLAQC-4 achieves the lowest compression rate. Moreover, the convergence performance of TLAQC surpasses that of QSGD with an 8-bit quantization level.

**Table 3:** MNIST.

Model	Q-level	Communication	Bits	cr	Accuracy
Full-precision^3^	–	1000	6.99 × ${10}^{8}$	1	98.24
QSGD^14^	8	1000	1.75 × ${10}^{8}$	25.03%	98.22
CosSGD^20^	8	1000	1.75 × ${10}^{8}$	25.07%	98.23
LAG^27^	–	459	3.21 × ${10}^{8}$	45.92%	98.19
TLAQC	8	535	9.40 × ${10}^{7}$	13.44%	98.39
	4	540	4.71 × ${10}^{7}$	6.73%	98.34

**Figure 5:**
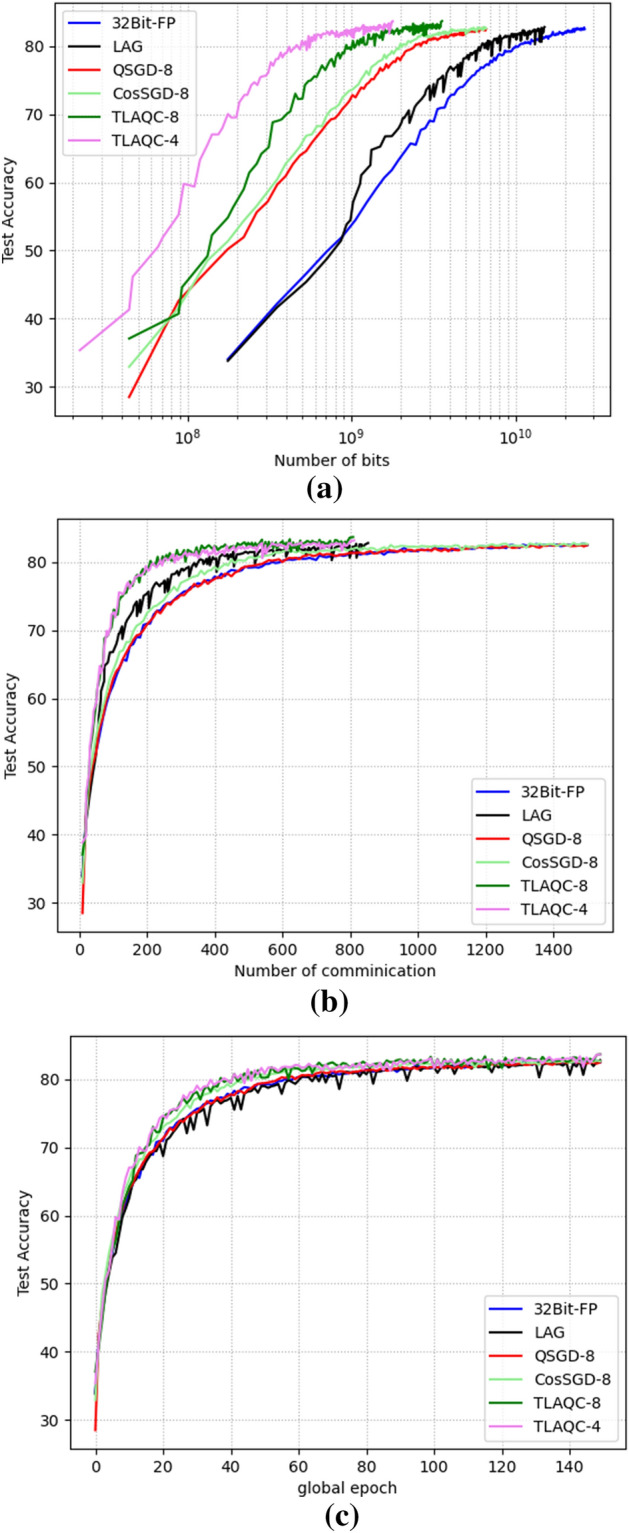
Comparision on CIFAR10 ((**a**) accuracy versus bits; (**b**) accuracy versus communication; (**c**) accuracy versus global epoch).

The CIFAR10 dataset has a larger number of model parameters compared to MNIST. In Fig. 5, we demonstrate the effectiveness of our proposed algorithm (TLAQC) on CIFAR10. We evaluate TLAQC under conditions of equal communication cost, equal communication rounds, and equal training rounds and observe superior convergence compared to other algorithms. Notably, the advantages of TLAQC's two-layer accumulation and quantization correction become more pronounced on more complex datasets. Table 4 further demonstrates that TLAQC achieves improved accuracy under the same number of global training rounds.

**Table 4:** CIFAR10.

Model	Q-level	Communication	Bits	cr	Accuracy
Full-precision^3^	–	1500	2.65 × ${10}^{10}$	1	82.4
QSGD^14^	8	1500	6.62 × ${10}^{9}$	25.01%	82.37
cosSGD^20^	8	1500	6.62 × ${10}^{9}$	25.01%	82.65
LAG^27^	–	702	1.24 × ${10}^{10}$	46.8%	82.93
TLAQC	8	804	3.55 × ${10}^{9}$	13.41%	83.65
	4	811	1.80 × ${10}^{9}$	6.76%	83.53

## Conclusion

The advantages of federated learning in protecting data privacy and addressing the problem of “data islands” have made it a crucial approach in various domains. To enhance the efficiency of federated learning and reduce communication costs, this paper introduces the communication-efficient TLAQC algorithm. TLAQC enables workers to train models using local data without sharing their private data with other nodes. While preserving node privacy, model training is accomplished by collectively uploading local training parameter information.

To achieve communication compression, TLAQC addresses two aspects. Firstly, a modified quantization method called RQSGD is proposed, and experimental results demonstrate its significant correction effect. Building upon quantization, TLAQC incorporates communication sparsification mechanisms by integrating a local self-inspection mechanism for workers, reducing the frequency of information uploads. To mitigate the impact of compression on model accuracy, TLAQC compensates for compressed model information through the double accumulation of quantization errors and retained weight deltas, all without incurring additional communication costs. By combining quantization methods and communication sparsification techniques while incorporating the accumulation of quantization errors and retained weight deltas, TLAQC greatly improves communication efficiency and exhibits exceptional algorithm performance.

There are still many imperfections in our work. This article only explores how to compress the uplink communication in federated learning. Later, we will explore the downlink compression method to improve the communication efficiency of federated learning further.

## Data Availability

The datasets analysed during the current study are available in the MNIST repository, http://yann.lecun.com/exdb/mnist/, and CIFAR10 repository, http://www.cs.toronto.edu/~kriz/cifar.html.
